# Sleep health and cognitive function among people with and without HIV: the use of different machine learning approaches

**DOI:** 10.1093/sleep/zsab035

**Published:** 2021-02-16

**Authors:** Davide De Francesco, Caroline A Sabin, Alan Winston, Michael N Rueschman, Nicki D Doyle, Jane Anderson, Jaime H Vera, Marta Boffito, Memory Sachikonye, Patrick W G Mallon, Lewis Haddow, Frank A Post, Susan Redline, Ken M Kunisaki

**Affiliations:** 1 Institute for Global Health, University College London, London, UK; 2 Department of Infectious Disease, Imperial College London, London, UK; 3 Brigham and Women’s Hospital, Boston, MA, USA; 4 Harvard Medical School, Harvard University, Boston, MA, USA; 5 Homerton University Hospital, London, UK; 6 Brighton and Sussex Medical School, Brighton, UK; 7 Chelsea and Westminster Healthcare NHS Foundation Trust, London, UK; 8 UK Community Advisory Board (UK-CAB), London, UK; 9 University College Dublin School of Medicine, Dublin, Ireland; 10 Kingston Hospital NHS Foundation Trust, London, UK; 11 King’s College Hospital NHS Foundation Trust, London, UK; 12 Beth Israel Deaconess Medical Center, Boston, MA, USA; 13 Minneapolis Veterans Affairs Health Care System, Minneapolis, MN, USA; 14 University of Minnesota, Minneapolis, MN, USA

**Keywords:** HIV, sleep, cognition, sleep quality, machine learning

## Abstract

**Study Objectives:**

We investigated associations between actigraphy-assessed sleep measures and cognitive function in people with and without HIV using different analytical approaches to better understand these associations and highlight differences in results obtained by these approaches.

**Methods:**

Cognitive and 7-day/night actigraphy data were collected from people with HIV (PWH) and lifestyle-similar HIV-negative individuals from HIV and sexual health clinics in the United Kingdom/Ireland. A global cognitive T-score was obtained averaging the standardized individual cognitive test scores accounting for sociodemographics. Average and *SD* of 11 sleep measures over 7 days/nights were obtained. Rank regression, partial least-squares (PLS) regression, random forest, sleep dimension construct, and latent class analysis (LCA) were applied to evaluate associations between global T-scores and sleep measures.

**Results:**

In 344 PWH (median age 57 years, 86% males), average sleep duration, efficiency, and wake after sleep onset were not associated with global T-scores according to rank regression (*p* = 0.51, *p* = 0.09, *p* = 0.16, respectively). In contrast, global T-scores were associated with average and *SD* of length of nocturnal awakenings, *SD* of maintenance efficiency, and average out-of-bed time when analyzed by PLS regression and random forest. No associations were found when using sleep dimensions or LCA. Overall, findings observed in PWH were similar to those seen in HIV-negative individuals (median age 61 years, 67% males).

**Conclusions:**

Using multivariable analytical approaches, measures of sleep continuity, timing, and regularity were associated with cognitive performance in PWH, supporting the utility of newer methods of incorporating multiple standard and novel measures of sleep-wake patterns in the assessment of health and functioning.

**Statement of Significance**

This is the first study to apply different machine learning approaches to assess the link between sleep and cognition in people with HIV, who are considered to be at high risk for both sleep and cognitive disorders.

## Introduction

Widespread access to combination antiretroviral therapy has meant that HIV is now a manageable chronic disease for many of those affected [1]. Nevertheless, the quality of life of people with HIV (PWH) remains poorer than that of the general population, in part due to an increased burden of co-morbidity [2], including highly prevalent and diverse sleep problems [3]. Established etiological pathways and risk factors for sleep problems in the general population may be exacerbated in PWH by the additional prevalence of several psychosocial and behavioral factors also known to disrupt sleep (e.g. depression, stress, excessive worry [4, 5]), by inflammation and neuronal damage induced by HIV [6] and by adverse effects of antiretroviral therapy [7].

Cognitive disorders also remain prevalent among PWH, especially mild or moderate disorders [8], with a reported prevalence often exceeding that seen in HIV-negative populations [9]. Sleep deprivation and/or fragmentation can be responsible for alterations of toxin clearance and synaptic function, potentially contributing to impairment of many cognitive functions [10]. While several studies have demonstrated the negative effect of poor self-reported [11] and objective [12, 13] sleep health on cognitive function of adults from the general population, little is known regarding the association between sleep and cognition in PWH, with limited evidence suggesting a link between poor self-reported sleep quality and cognitive disorders. However, the few studies on PWH that have investigated these associations either relied on limited sample size [14], on self-reported sleep quality [15], or lacked a control group of HIV-negative individuals [16]. Moreover, most of the studies that have investigated the link between sleep and cognition in the general population have either considered self-reported measures of sleep [11] or have focused on sleep duration only [12, 13], with only a few investigating the role of multiple dimensions of sleep [17–21]. Thus, information on associations between objective measures of sleep and cognitive function is lacking.

The idea of sleep health as a multidimensional construct [22] recognizes that different aspects of sleep (e.g. duration, efficiency, timing, and regularity) may all be important and have differential effects on health. Therefore, investigation of a single sleep characteristic only (e.g. sleep duration) may lead to a limited understanding of the broader implications of poor sleep health. The introduction of accelerometers in clinical research has allowed researchers to objectively quantify various aspects of sleep over a prolonged period of time. Recently, there has been increasing interest in using a variety of statistical methods to exploit these data for studying the effect of sleep on health outcomes [23]. Several studies have begun to consider multiple sleep characteristics using multivariate analytical methods, such as random forests, to handle high-dimensional and correlated sleep data [24]. Other studies have examined sleep as a multidimensional construct and have described the association of a composite sleep score or individual sleep dimensions on a given outcome [25, 26]. Individual-based approaches have also been applied, such as cluster and latent class analysis (LCA), to identify sleep health “profiles” based on several sleep characteristics and then investigate differences in health outcomes across the different “profiles” [27].

These are conceptually different approaches each of which can provide important insight into the association between sleep and health outcomes. However, intrinsic differences related to the nature of these approaches and their specific aims can potentially lead to different conclusions about the relative importance of each sleep characteristic or dimension for a given outcome.

Our overarching aim was to assess the association between sleep health and cognitive function in both PWH and HIV-negative controls and shed light on those aspects of sleep most strongly linked to cognition. While addressing this study question, we sought to evaluate several different statistical approaches that differ in how they take into account the relationships between sleep parameters. Secondly, when possible, we investigated the extent to which associations between sleep health and cognitive function may differ by HIV status.

## Methods

### Study participants and procedures

The Pharmacokinetic and Clinical Observations in People Over Fifty (POPPY) study is an observational cohort study of PWH and HIV-negative individuals with similar lifestyles from the United Kingdom and Ireland. The study recruited three groups of individuals and has been described previously [28]. Briefly, two groups of PWH were recruited from eight HIV clinics: PWH aged at least 50 years and PWH aged 18–50 years, with the latter group frequency-matched on gender, ethnicity, sexual orientation, and location to the older group of PWH. Inclusion criteria were documented presence of HIV infection, white or black-African ethnicity, likely route of HIV acquisition via sexual exposure, and ability to comprehend the study information leaflet. A group of HIV-negative individuals was also recruited from sexual health centers affiliated to the HIV clinics. These individuals were required to be 50 years or older and to have a documented negative HIV test. In addition, this group was frequency-matched to the group of PWH aged at least 50 years on gender, ethnicity, sexual orientation, and location.

Subsets of POPPY study participants from the three groups were recruited into this nested substudy, without regard to sleep symptoms. Additional inclusion criteria were the ability to wear a fingertip oximetry device and wrist actigraph for a week and to adhere to study procedures (according to the investigator’s judgment).

Participants underwent a single study visit between March 2017 and July 2018 followed by in-home procedures including a daily sleep diary, actigraph and oximetry measurements, and an additional visit to return the devices and the completed diaries. At the study visit, participants completed questionnaires detailing sleep quality, symptoms of sleep disorders, sleep medical history, and medication use for sleep disorders and underwent detailed assessment of anthropometric measurements and cognitive function. All participants provided written informed consent and the study was approved by the UK Research Ethics Committee (Fulham, London; UK number 16/LO/1409) and local ethics committees and/or institutional review boards. For the present analysis, only participants with at least 5 days/nights’ worth of valid actigraphy data and with completed cognitive assessment were included.

### Cognitive function

Participants underwent a detailed assessment of cognitive function using a comprehensive battery of nine tests covering five domains known to be affected by HIV-associated cognitive impairment: language, attention, processing speed, executive function, and motor function (Supplementary Table 1) [8]. The battery was administered by trained research staff. Individual test scores were converted into T‐scores (mean of 50 and *SD* of 10) using appropriate normative data accounting for age, gender, ethnicity, and education as appropriate. Individual test T-scores were averaged to obtain domain T-scores which were, in turn, averaged to obtain a global T-score of cognitive function. Higher T-scores indicate better cognitive function.

### Actigraphy data

A triaxial actigraph device (ActiGraph wGT3X-BT; ActiGraph Corporation) was used to record activity data and estimate sleep parameters. Actigraphs were programmed to collect data at a sampling rate of 100 Hz and participants were instructed to wear the device on the nondominant wrist continuously until the time of return (a minimum of 7 days later), with removal only when needed to avoid damage to the device (e.g. contact sports, swimming, bathing). In addition, daily sleep diaries were completed at home by study participants describing the timing of sleep, nocturnal awakenings, daytime napping, and interruptions in the use of the actigraph device.

Upon return of the device to study sites, data from the devices were downloaded at an epoch length of 15 s. After successful download, digital data files were transferred to the sleep reading center for central scoring. For each recording day, the sleep (or “rest”) periods were manually annotated based on a combination of sleep diary data (reporting bed and wake times) and visualization of an abrupt decrease (<1,000 counts) and increase (≥1,000 counts) in activity for 5 or more minutes, respectively. Daytime naps were scored based on identifying periods reported in the sleep diary as naps, accompanied (within 30 min) by decreased activity. Sleep-wake epochs were then identified using the Cole–Kripke algorithm [29]. Daily sleep measures were obtained including sleep onset, midpoint (clock time between sleep onset and offset) and out-of-bed time, onset latency, duration (total time spent asleep), wake duration after sleep onset (WASO), maintenance efficiency (% of time spent asleep from sleep onset and sleep offset), movement index (% of 60-s epochs with movement divided by time spent in bed in hours), fragmentation index (% of 60‐s sleep epochs out of the total number of epochs in the sleep period), and number and length of nocturnal awakenings. For each participant, the average and the within-individual variability (i.e. the *SD*) across the observation period were obtained for each of these measures.

### Sleep questionnaires

Sleep questionnaires were administered at study visits, including the Insomnia Severity Index [30] and the Patient-Reported Outcomes Measurement Information System sleep disturbance and sleep-related impairment questionnaires [31]. In particular, answers to questions related to participants’ satisfaction with their current sleep from these two questionnaires were analyzed: “How satisfied/dissatisfied are you with your current sleep pattern?” and “I was satisfied with my sleep,” respectively.

### Sleep dimensions

Six dimensions of sleep health as proposed by Buysse [22], i.e. satisfaction, alertness, timing, efficiency, duration, and regularity (the so-called RU SATED scale [32]), were derived from the daily actigraphy measures and questionnaire data. Scores for the dimension of satisfaction were determined as follows: 2 (i.e. “good”) if answering “Very satisfied” or “Moderately satisfied” to the “How satisfied/dissatisfied are you with your current sleep pattern?” question and “Quite a lot” or “Very much” to the “I was satisfied with my sleep” question; 0 (i.e. “poor”) if answering “Dissatisfied” or “Very dissatisfied” and “Not at all” or “A little bit,” respectively, to the same questions; and 1 (i.e. “fair”) in all other circumstances.

In addition to continuous measures summarizing weekly averages and standard deviations, daily number of naps, sleep onset and out-of-bed time, maintenance efficiency, and duration as measured by the actigraphy device were used to derive dichotomous variables indicating “good” vs “bad” health for the dimensions of alertness, timing, efficiency, duration, and regularity. For each recorded night, “good” vs “bad” sleep health was defined as follows: 0 naps vs at least 1 naps (alertness), sleep onset time before 02:00 am and out-of-bed time after 04:00 am vs onset time after 02:00 am or out-of-bed time before 04:00 am (timing), maintenance efficiency at least 85% vs less than 85% (efficiency), sleep duration between 6 and 8 h vs less than 6 or more than 8 h (duration), and sleep onset time within 30 min of the average sleep onset time across the whole observation period vs onset time at least 30 min before or after the average onset time (regularity). Cutoffs were selected to reflect the dimensions as originally proposed by Buysse [22], with the exception of sleep efficiency for which a current recommendation was used [33]. For each dimension, a score of 2 (i.e. “good”) indicates at least 70% of recorded nights classified as “good,” 1 (i.e. “fair”) indicates between 30% and 70% of “good” nights, and 0 (i.e. “poor”) indicates less than 30% of “good” nights.

A total sleep health score was obtained by summing up the scores from the six dimensions; scores range from 0 to 12 with higher values indicating better sleep health.

### Statistical analysis

Continuous variables, including cognitive T-scores and actigraphy measures, were summarized using the median and the interquartile range (IQR); categorical variables were described using frequencies and percentages. Comparisons of sociodemographic, lifestyle, and clinical characteristics across older PWH, younger PWH, and HIV-negative individuals were carried out using chi-square test, Fisher’s exact test, and Wilcoxon rank‐sum tests as appropriate. The two groups of PWH were subsequently combined into a single group of PWH; comparisons of actigraphy measures and cognitive scores between PWH and HIV-negative individuals were performed using median regression, adjusting for age.

Different approaches to evaluate the association between actigraphy-assessed sleep measures and global cognitive function (i.e. the global T-score obtained as the average of the five domain T-scores) were applied. These approaches have been previously used to investigate the link between sleep and health outcomes, as also reviewed by Matricciani et al. [23]. A brief overview of these methods with advantages and disadvantages of each method is reported in Table 1; further details concerning their use in this study are given below.

**Table 1.** Overview of the analytical approaches used to investigate the association between sleep health and cognitive function

Analytical approach	Overview	Advantages	Disadvantages	Comments on statistical power
Traditional approach (multivariable regression analysis)	Multivariable regression analysis is used to describe the relationship between a set of independent variables (e.g. sleep parameters) and an outcome variable (e.g. a health outcome), through mathematical models (e.g. in linear regression the outcome is modeled as a linear combination of the independent variables).	It allows the estimation of independent relationships between sleep variables.	Due to issues related to the number and collinearity of sleep variables, its use is often limited to the inclusion of few preselected sleep variables, in order to avoid overfitting and instability in the estimation of model parameters.	Statistical power depends mainly, other than the sample size, on the number of independent variables. There are no generally agreed methods for relating the sample size versus the number of independent variables. Common rules-of-thumbs recommend at least 15 or 30 observations per variable [41, 42].
		It is the most widely used method to investigate associations. Due to its popularity, the interpretation of regression analysis is widely accessible to non-statisticians.		
			It often oversimplifies the relationships between the variables of interests and handling complex patterns of relationships is difficult.	
Partial least-squares (PLS) regression	PLS-based methods reduce the input variables (e.g. sleep variables) to latent variables and regress those latent variables against the outcome. Metrics such as the variable importance in prediction are then calculated to rank each of the input variables according to their importance to predict the outcome.	PLS regression is preferable to standard regression analysis when there are multiple input variables, and when these input variables are correlated.	It is often difficult to interpret the model parameters that define the latent variables and those that relate these to the outcome. Both the latent variables and the outcome are modeled as a linear combination (of the input variables and the latent variables, respectively). Therefore, nonlinear relationships would be missed.	PLS-based methods are thought to provide significant advantages when analyzing small sample sizes or data with a small number of observations to the number of variables ratios. However, the optimal approach to assess statistical power for PLS-based approaches is still debated [43].
			It only handles continuous input variables and does not provide a straightforward way to account for potential confounders and effect modifiers (e.g. HIV-status).	
				Statistical power should be determined based on various factors, such as distributional assumptions, characteristics of the input variables, or the strength of the relationships of interest [44].
Random forest	Random forest is a nonparametric, multivariable ensemble technique based on decision trees. Several d ifferent decision trees, each randomly selecting a subset of observations and input variables, are merged into one learner.	The random selection of sampled observations and input variables helps the model to avoid overfitting.	It can be computationally intense and require longer times to train.	Random forest has been reported to be robust to model training with small sample sizes [45].
			The estimated relationship between the outcome and the input variables can be difficult to interpret with no direct measure to evaluate either the direction or magnitude of associations.	
				In particular, it has been shown to perform well in terms of statistical power, when the distributions of input variables were skewed [46].
		It can handle both categorical and quantitative variables and deal with missing data.		
		It requires minimal assumptions about the type of associations between the outcome and the input variables and can detect nonlinear associations.		
Multidimensional construct	Objective or self-reported sleep parameters are combined using predefined criteria to derive several sleep dimensions. Associations between these sleep dimensions are then investigated, typically using regression-based approaches.	It allows to integrate clinical knowledge and expert opinion into the analysis.	There is not an objectively defined paradigm that describes which are the dimensions that characterize sleep health and how to best define and operationalize each dimension.	Statistical power depends on the statistical method used to investigate associations between the outcome and the sleep dimensions. When using regression, the use of fewer sleep dimensions, compared to the individual sleep variables, improves statistical power.
Latent class analysis (LCA)	LCA helps recognizing latent sleep profiles that are shared by many individuals who, in turn, may experience similar risks for health outcomes. Latent sleep profiles refer to the specific combinations of several sleep characteristics experienced by individuals.	It is flexible with respect to the distribution of sleep variables.	The identification of sleep profiles is not oriented toward the assessment of relationships with health outcomes.	Little is known about the exact effect of sample size on the ability to identify the set of underlying latent profiles. Simulations have shown that having a too small sample size often leads to choosing too few latent profiles to adequately describe the data-generating model [47].
		It can accommodate different data types, including non-normal and skewed continuous sleep variables.	The selection of the appropriate number and the underlying distribution of profiles is often challenging.

1. Traditional approach: We investigated individual sleep measures selected based on prior knowledge and hypothesized pathological mechanisms [34, 35], that is the average sleep duration, maintenance efficiency, and WASO. Associations with global cognitive T-scores were evaluated using rank regression to account for the skewness of variables of interest, adjusting for potential confounders such as age, gender, ethnicity, education, and use of sleep medication. For sleep duration, where a U-shaped relationship with cognitive function can be expected, we also evaluated the absolute value of the difference between the observed duration and the median sleep duration (i.e. 7 h). Each sleep measure was analyzed independently (using separate regression models) and also simultaneously in a single regression model also including potential confounders. Analyses were conducted separately in PWH (older and younger PWH combined) and HIV-negative individuals; the interaction between HIV status and each sleep measure was tested using rank regression (also including potential confounders) to evaluate differences in the associations between the two groups.
2. Partial least-squares (PLS) regression [36]: This multivariate approach was applied separately in PWH and HIV-negative individuals, to the averages and SDs of all continuous sleep measures (sleep onset, midpoint and out-of-bed time, onset latency, duration, WASO, maintenance efficiency, movement index, fragmentation index, number and length of nocturnal awakenings, i.e. a total of 22 variables) to predict global cognitive T-scores. All variables were centered and scaled to unit variance. For each variable, the variable importance for prediction (VIP) was calculated as a measure of the strength of the association between that variable and the global T-score [37] and in order to rank sleep measures with respect to their association with cognitive function.
3. Random forest [38]: This multivariate approach was also applied separately in PWH and HIV-negative controls, with sleep measures (both the average and *SD*) and potential confounders (age, gender, ethnicity, education, and use of sleep medication) as inputs (a total of 22 sleep measures plus 5 covariates) and the global cognitive T-score as the outcome. Among PWH (HIV-negative individuals), the random forest approach fitted 5,000 (8,200) regression trees, each of which utilized 15 (5) randomly chosen variables of the 27 input variables (see also Supplementary Material and Supplementary Figure 1) and empirically selected those that optimally split the sample into two subgroups with global T-scores that were as different as possible. For each input variable, the variable importance measure (VIM) indicates the total decrease in the residual sum of squares from splitting on that variable, averaged over all trees. VIM was used to rank input variables in terms of their ability to predict the global T-score; variables with larger VIM have greater predictive ability. In addition, the importance of each variable was assessed by comparing the reduction in the *R*^2^ consequent to the exclusion of that variable from the model.
4. Sleep health as a multidimensional construct: We investigated associations of each sleep dimension and their sum in the previously proposed six-item RU SATED scale with global cognitive T-scores. Associations were investigated, separately in PWH and HIV-negative individuals, using median regression to test differences in overall cognitive function between individuals reporting good, fair, and poor sleep in each dimension, when there were at least five individuals in a given group. The six dimensions were considered individually in separate models. The association between global T-scores and the total sleep health score obtained as the sum of the six dimensions was assessed using rank regression. In all the regression models, we adjusted for age, gender, ethnicity, education, and use of sleep medication.
5. Latent class analysis [39]: This individual-based approach was used to identify distinct groups of PWH and, separately, of HIV-negative individuals on the basis of the observed sleep measures (both averages and *SD*s) centered and scaled to unit variance. Groups were identified using a model-based clustering algorithm based on parameterized finite Gaussian mixture models [40]. The optimal number of groups was selected using the Bayesian Information Criterion (BIC) and was evaluated using the bootstrap likelihood ratio test and appropriate stability measures. In order to interpret the obtained groups, means and 95% confidence intervals (CIs) of each sleep measure were obtained within each identified group. Median regression was used to evaluate the difference in global cognitive T-scores between groups returned by the LCA, while adjusting for age, gender, ethnicity, education, and use of sleep medication.

Additional information regarding hyper-parameter optimization, validation procedures, and the predictive performance of PLS regression, random forest, and LCA are reported in Supplementary Material. Analyses were performed using the statistical software R, version 3.6.0 and the libraries “quantreg,” “ropls,” “randomForest,” and “mclust.”

## Results

### Participant characteristics

A total of 241 older PWH, 103 younger PWH, and 119 HIV-negative individuals completed the cognitive battery and had at least 5 days/nights of actigraphy data (Table 2). Compared to the HIV-negative individuals, PWH were more likely to be male (86.3% vs 67.2%, *p* < 0.001), men who have sex with men (79.1% vs 52.9%, *p* < 0.001), be retired or not working (48.3% vs 37.1%, *p* = 0.006) and to report ongoing use of sleep medication (8.7% vs 1.7%, *p* = 0.006), current smoking (26.2% vs 15.1%, *p* = 0.01), recreational drug (26.7% vs 14.3%, *p* = 0.006), and current/previous injection drug use (9.3% vs 1.7%, *p* = 0.004). Current alcohol use was more frequent among HIV-negative individuals compared to older PWH (91.6% vs 81.1%, *p* = 0.007).

**Table 2.** Sociodemographic, lifestyle, clinical, and HIV-related characteristics of study participants

Median (IQR) or *n* (%)	PWH (*n* = 344)	HIV negative (*n* = 119)	*p*
Male gender	297 (86.3%)	80 (67.2%)	<0.001
Age (years)	57 (52–62)	61 (57–66)	<0.001
White ethnicity	305 (88.7%)	109 (91.6%)	0.37
MSM/homosexual	272 (79.1%)	63 (52.9%)	<0.001
University degree or above	158 (45.9%)	61 (51.3%)	0.32
Years of education	16 (12–18)	16 (13–18)	0.76
BMI (kg/cm^2^)	25.4 (23.5–28.7)	25.8 (23.6–29.7)	0.18
Resting pulse oximetry (mm Hg)	96 (95–98)	96 (95–97)	0.68
Use of sleep medication*	30 (8.7%)	2 (1.7%)	0.006
Work schedule			0.006
Day shift	120 (35.1%)	60 (51.7%)	
Other/irregular shift	57 (16.7%)	13 (11.2%)	
Retired/do not work	165 (48.3%)	43 (37.1%)	
Current alcohol use	279 (81.1%)	109 (91.6%)	0.007
Current smoking	90 (26.2%)	18 (15.1%)	0.01
Current recreational drugs	92 (26.7%)	17 (14.3%)	0.006
Ever injected drugs	32 (9.3%)	2 (1.7%)	0.004
Current CD4^+^ count (cells/µL)	630 (483–835)	N/A	N/A
Nadir CD4+ count (cells/µL)	190 (87–290)	N/A	N/A
Years since HIV diagnosis	17.6 (10.8–24.6)	N/A	N/A
On antiretroviral treatment	316 (91.9%)	N/A	N/A
HIV RNA <40 copies/mL	332 (97.1%)	N/A	N/A
Global T-score	50.0 (44.2–54.9)	52.0 (48.3–55.6)	0.01
Sleep duration (h)	7.0 (6.3–7.6)	7.2 (6.7–7.6)	0.12
Maintenance efficiency (%)	88.7 (84.4–91.4)	90.2 (86.2–92.2)	0.01
WASO (min)	54 (40–74)	49 (35–69)	0.02

PWH, people with HIV; MSM, men who have sex with other men.

*These include Amitriptyline (5 PWH), Nitrazepam (2 PWH), Nytol (2 PWH), Zopiclone (13 PWH and 1 HIV-negative), Diazepam (2 PWH), and other medications.

PWH had been diagnosed with HIV for a median (IQR) of 17.6 (10.8–24.6) years, 97.1% had an HIV RNA less than 40 copies/mL, and the median (IQR) CD4^+^ cell count was 630 (483–835) cells/μL.

### Cognitive scores and sleep measures

Median (IQR) global T-score was 50.7 (44.3–55.4) in older PWH, 48.7 (43.0–54.0) in younger PWH (*p* = 0.15 compared to older PWH), and 52.0 (48.3–55.6) in HIV-negative individuals (*p* = 0.04 compared to older PWH). When combining older and younger PWH, global T-scores did not differ significantly from those of HIV-negative individuals (median [IQR] in the combined group of PWH was 50.0 [44.2–54.9], *p* = 0.21 after adjusting for age; Supplementary Table 2).

Correlations between actigraphy variables are given in Supplementary Table 3. There were no differences between PWH and HIV-negative individuals, after adjusting for age, with regard to average sleep onset time, onset latency, duration, WASO, fragmentation index, and number of nocturnal awakenings (Supplementary Table 2). PWH were observed to have later average sleep mid-point and out-of-bed times compared to HIV-negative individuals (*p* = 0.01 and *p* = 0.02, respectively). The average movement index (%) was greater in PWH compared to HIV-negative individuals (median [IQR]: 17.7 [14.3–22.5] vs 15.2 [12.5–19.6], *p* = 0.003), with a tendency toward lower average maintenance efficiency (*p* = 0.07) and greater mean length of awakenings (*p* = 0.08) in PWH compared to HIV-negative individuals.

There were significant differences between groups with respect to the night-to-night variability of sleep onset time (*p* = 0.05), duration (*p* = 0.01), and movement index (*p* = 0.04), with weaker evidence regarding fragmentation index (*p* = 0.07) and mean length of nocturnal awakenings (*p* = 0.08). For all these measures, there was greater night-to-night variability in PWH compared to HIV-negative individuals.

Finally, PWH were classified as having poorer overall sleep health as measured by the RU SATED construct: Median (IQR) sleep health score was 7 (6–9) in PWH and 8 (7–10) in HIV-negative individuals (*p* < 0.001 after adjustment for age, Supplementary Table 2). In particular, PWH had poorer sleep health than HIV-negative individuals in the satisfaction (*p* = 0.008), timing (*p* ≤ 0.001), and duration (*p* = 0.001) dimensions (Supplementary Table 4). Weaker evidence suggests that PWH experienced poorer health in the other sleep dimensions: alertness, efficiency, and regularity, although only the associations with regularity met the strict threshold for statistical significance (*p* = 0.07, *p* = 0.07, and *p* = 0.05 for the three dimensions, respectively).

### Association between sleep health and overall cognitive function

#### Traditional approach

Among PWH, global cognitive T-scores were not associated with average sleep duration (adjusted rho = 0.08, *p* = 0.12), WASO (adjusted rho = −0.03, *p* = 0.55), or maintenance efficiency (adjusted rho = 0.06, *p* = 0.25, after adjustment for potential confounders; Table 3). While there was no strong evidence of an association with the absolute value of the median-centered sleep duration (*p* = 0.36) in PWH, longer/shorter sleep duration was associated with poorer cognitive function in HIV-negative individuals (adjusted rho = −0.21, *p* = 0.02). Among HIV-negative individuals, higher WASO (adjusted rho = 0.16) and lower maintenance efficiency (adjusted rho = −0.16) were associated with better cognitive function, although associations did not reach statistical significance (*p* = 0.08 and *p* = 0.07, respectively). However, observed associations did not significantly differ between PWH and HIV-negative individuals (all *p*s for the interaction term >0.05).

**Table 3.** Association of average sleep duration, WASO, and maintenance efficiency with the global cognitive T-score, as estimated via rank regression, adjusting for age, gender, ethnicity, education, and ongoing use of sleep medication

Sleep measure	PWH (*n* = 344)		HIV negative (*n* = 119)		*p* interaction
	Adj. rho (95% CI)	*p*	Adj. rho (95% CI)	*p*	
*Univariable analysis*					
Average duration	0.08 (−0.02 to 0.17)	0.12	−0.02 (−0.20 to 0.15)	0.78	0.49
Absolute value of median-centered average duration	−0.04 (−0.14 to 0.05)	0.36	−0.21 (−0.38 to −0.04)	0.02	0.71
Average WASO	−0.03 (−0.13 to 0.07)	0.55	0.16 (−0.02 to 0.34)	0.08	0.27
Average maintenance efficiency	0.06 (−0.04 to 0.16)	0.25	−0.16 (−0.35 to 0.01)	0.07	0.18
*Multivariable analysis*					
Absolute value of median-centered average duration	−0.03 (−0.13 to 0.06)	0.51	−0.25 (−0.43 to −0.07)	0.008	0.64
Average WASO	0.21 (−0.09 to 0.51)	0.16	0.27 (−0.51 to 1.06)	0.49	0.71
Average maintenance efficiency	0.26 (−0.04 to 0.56)	0.09	0.08 (−0.71 to 0.86)	0.85	0.50

In multivariable analysis, maintenance efficiency showed the strongest association with the global T-score in PWH but without reaching statistical significance (adjusted rho = 0.26, *p* = 0.09), with greater efficiency being associated with better cognitive scores. Among HIV-negative individuals, sleep duration remained significantly associated with global T-scores (adjusted rho of −0.25, *p* = 0.008), with no associations for WASO and maintenance efficiency (*p* = 0.49 and *p* = 0.85, respectively; Table 3).

#### Multivariate approach: PLS

**Figure 1.**
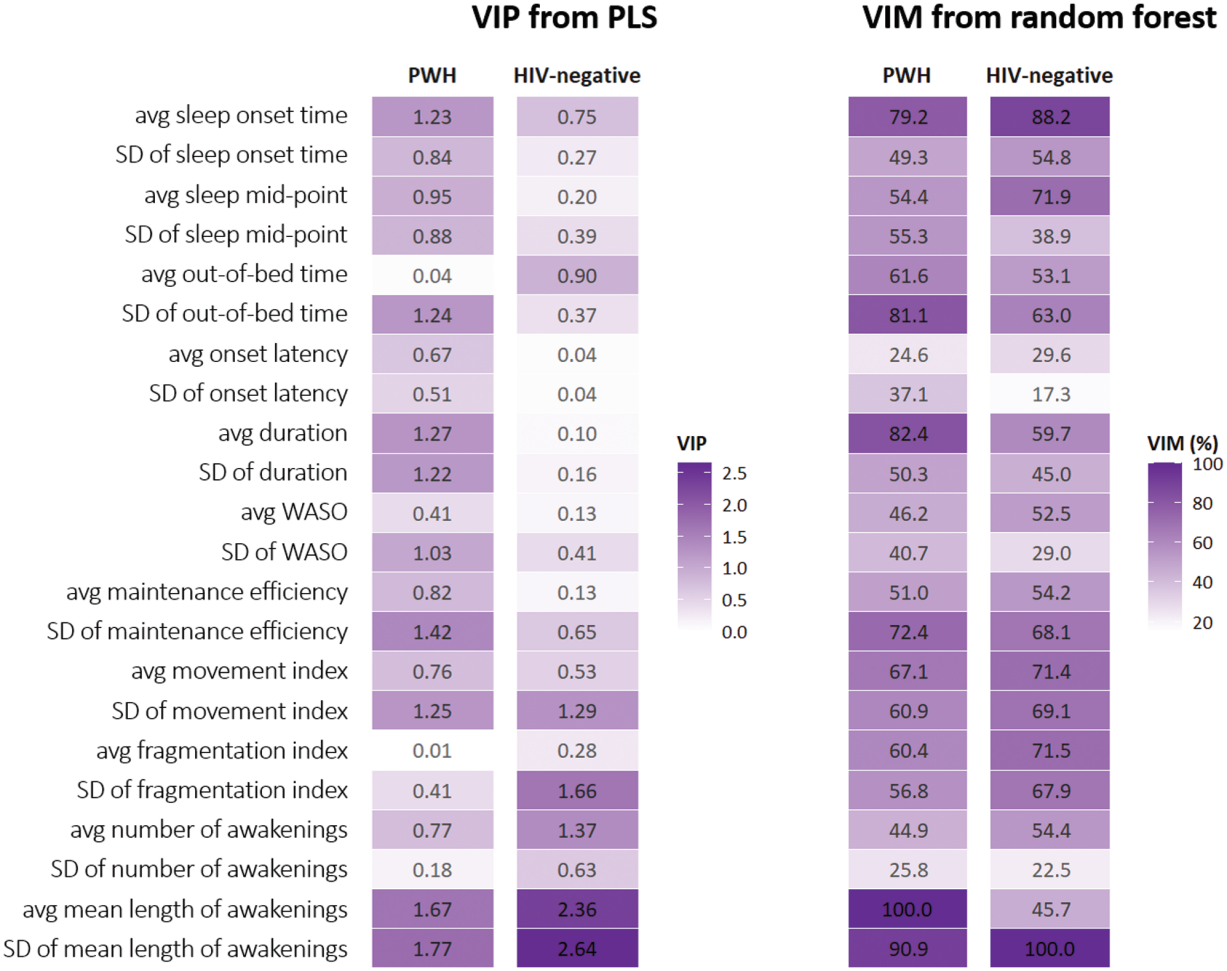
VIP and VIM for each actigraphy variable as obtained from PLS and random forest, respectively, run separately in PWH (*n* = 344) and HIV-negative individuals (*n* = 119). *Note*: VIM is expressed as the percentage relative to that of the variable with the highest VIM. The random forest model additionally included age, gender, ethnicity, education, and use of sleep medication; VIM for all variables, including potential confounders, are shown in Supplementary Figure 2.

Among PWH, PLS extracted one predictive component (PLS score) obtained as a linear combination of the 22 actigraphy variables. This component explained 40.6% of the total variance in the actigraphy variables and was significantly correlated with the global T-score (rho = 0.30 [0.20–0.39], *p* < 0.001). The variables that contributed the most to the PLS score were *SD* of mean length of awakenings (VIP = 1.77), average of mean length of awakenings (VIP = 1.67), *SD* of maintenance efficiency (VIP = 1.45), average duration (VIP = 1.27), *SD* of movement index (VIP = 1.25), *SD* of out-of-bed time (VIP = 1.24), average sleep onset time (VIP = 1.23), and *SD* of duration (VIP = 1.22, Figure 1). Specifically, as indicated by negative weights, greater variability in mean length of awakenings, maintenance efficiency, movement index, out-of-bed time and duration, and later average onset time were all associated with poorer cognitive scores. Longer average sleep duration (positive weight) was associated with better scores.

Among HIV-negative individuals, one component was extracted, explaining 38.5% of the total variance in the actigraphy variables. The component was significantly correlated with the global T-score (rho = 0.38 [0.22–0.52], *p* < 0.001), and VIP was the highest for *SD* of mean length of awakenings (VIP = 2.64), average of mean length of awakenings (VIP = 2.36), *SD* of fragmentation index (VIP = 1.66), average number of awakenings (VIP = 1.37), and *SD* of movement index (VIP = 1.29). Based on the sign of the respective weights, greater variability of mean length of awakenings, fragmentation index and movement index, and longer average of mean length of awakenings were associated with poorer cognitive scores. A greater average number of awakenings was linked to better cognitive scores.

#### Multivariate approach: random forest

The VIMs for the 22 actigraphy variables derived from random forest and expressed as the percentage relative to the variable with the highest VIM are reported in Figure 1. Among PWH, the average mean length of awakenings showed the highest VIM among all the actigraphy variables (the corresponding average decrease in residual sum of squares was 1,133.1) to separate PWH with different cognitive scores. VIM was also high for the *SD* of mean length of awakenings (90.9% of VIM of average mean length of awakenings), average sleep duration (82.4%), *SD* of out-of-bed time (81.1%), and average sleep onset time (79.2%).

Among HIV-negative individuals, the *SD* of mean length of awakenings showed the greatest ability to predict global cognitive scores, with an average decrease in residual sum of squares of 284.6. VIM for the average sleep onset time, sleep mid-point, fragmentation index, and movement index were 88.2%, 71.9%, 71.5%, and 71.4%, respectively of the VIM of the *SD* of mean length of awakenings.

#### Sleep health as a multidimensional construct

**Figure 2.**
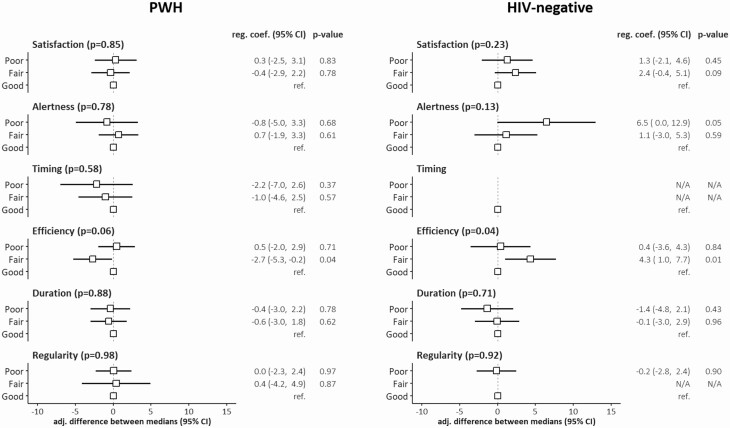
Association between RU SATED sleep dimensions and global cognitive T-score in PWH (*n* = 344) and HIV-negative individuals (*n* = 119). Associations are expressed as differences in the median global T-scores with participants reporting “good” sleep health as a reference category and adjusting for potential confounders.

None of the six sleep dimensions (satisfaction, alertness, timing, efficiency, duration, and regularity) was individually significantly associated with the overall cognitive function of PWH (Figure 2). Efficiency was the only dimension approaching statistical significance (*p* = 0.06) with cognitive scores; PWH with fair efficiency (i.e. poor efficiency during 30%–70% of nights) showed poorer cognitive scores than PWH with good sleep efficiency (adjusted difference in global T-score [95% CI] = −2.7 [−5.3 to −0.2], *p* = 0.04). For all other dimensions, the median global T-score did not appear to differ significantly across PWH with poor, fair, or good sleep health, after adjustment for potential confounders.

Among HIV-negative individuals, sleep efficiency appeared to be associated with overall cognitive function (*p* = 0.04), with individuals reporting fair efficiency showing better cognitive scores than those reporting high efficiency (adjusted difference in global T-score [95% CI] = 4.3 [1.0–7.7], *p* = 0.01). This association appeared to differ from that seen in PWH (*p* for interaction = 0.002). Associations of other sleep dimensions with the global T-score were weak and nonsignificant (Figure 2) and did not differ significantly from associations observed in PWH (interaction *p* = 0.16 for satisfaction, *p* = 0.96 for alertness, *p* = 0.85 for duration, and *p* = 0.88 for regularity).

Finally, the association of the total sleep health score with overall cognitive function was weak in both PWH and HIV-negative individuals (adjusted rho [95% CI] = 0.04 [−0.06 to 0.14], *p* = 0.42 and −0.05 [−0.24 to 0.13], *p* = 0.57, respectively). There was also no evidence that the association differed by HIV status (*p* for interaction = 0.25).

#### Individual-based approach: LCA

**Figure 3.**
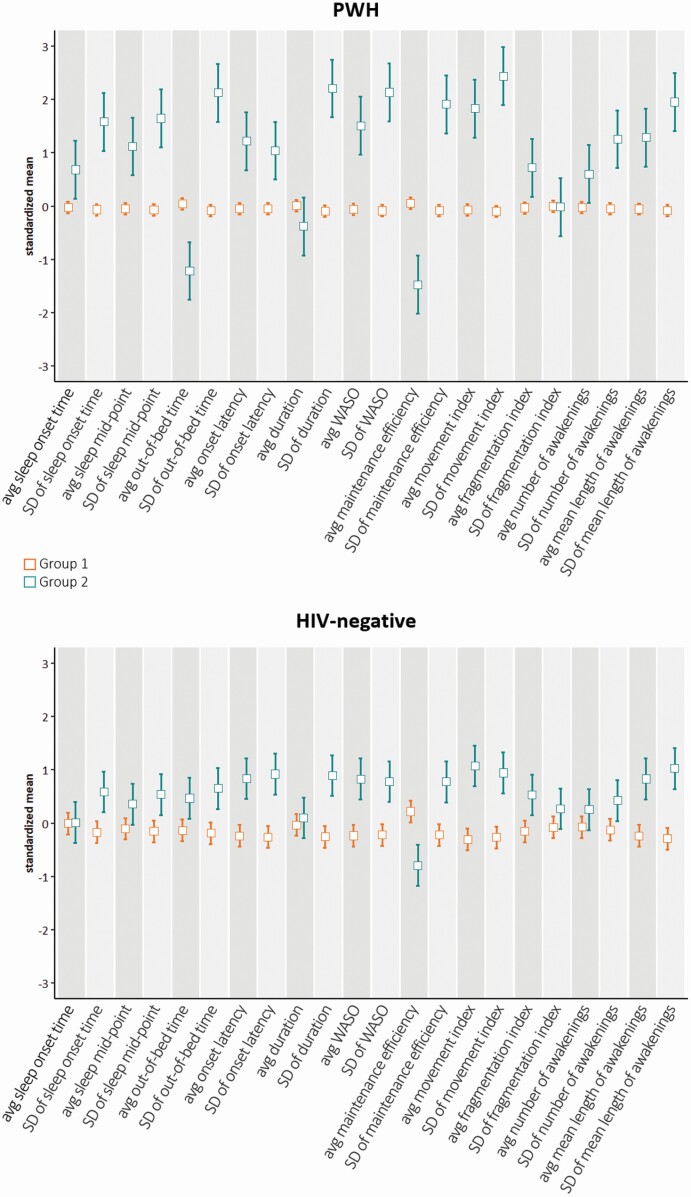
Means (95% CI) of the 22 sleep variables (centered and scaled to unit variance) in PWH and HIV-negative individuals, stratified by groups identified using LCA.

According to the BIC and stability measures (Supplementary Figures 4 and 5), a model with two latent groups was identified based on sleep parameters observed in PWH. The mean and 95% CI of the 22 actigraphy-assessed sleep variables in PWH allocated to the two groups are shown in Figure 3. Group 1 included the majority of PWH (*n* = 331, 96.2%) with only 13 (3.8%) PWH in Group 2. Compared to PWH in Group 1, those in Group 2 had later average time of sleep onset and mid-point, earlier average time of out-of-bed, longer average onset latency and WASO, shorter average duration, poorer average maintenance efficiency, greater average movement index, fragmentation index, number, and length of nocturnal awakenings. Variability of all sleep measures except fragmentation index was greater among PWH in Group 2 compared to those in Group 1. The median (IQR) global T-score was 50.0 (44.2–54.9) and 50.0 (45.7–52.4) in PWH in Groups 1 and 2, respectively. After adjustment for potential confounders, the difference between the two groups was not significant (adjusted difference in global T-score [95% CI] = 1.4 [−2.4 to 4.0], *p* = 0.59).

Two groups were also identified by the LCA in HIV-negative individuals (Figure 3). Compared to individuals in Group 1 (*n* = 93, 78.2%), those in Group 2 (*n* = 26, 21.8%) showed later average mid-point and out-of-bed time, longer average onset latency and WASO, lower average maintenance efficiency, greater average movement, fragmentation, and mean length of awakenings. In Group 2, the night-to-night variability of all the variables except for fragmentation index was greater compared to that observed in individuals in Group 1. The median (IQR) global T-score was 52.9 (48.4–55.9) and 51.5 (47.7–54.7) in HIV-negative individuals in Groups 1 and 2, respectively. These did not differ significantly after controlling for potential confounders (adjusted difference in global T-score [95% CI] = 0.1 [−2.7, 2.7], *p* = 0.97).

## Discussion

This is the first study, to our knowledge, that has comprehensively assessed the relationships between objectively measured, actigraphy-assessed sleep characteristics, and cognitive function in a multicenter cohort of PWH, with an appropriate control group. We also applied several analytical approaches to these complex, correlated, and multidimensional actigraphy data and found that interpretation of results differed by the analytic approach used, highlighting the importance of more advanced machine learning approaches to better handle these complex data and shed light on the impact of sleep on health outcomes.

When we applied the traditional approach of investigating individual sleep characteristics hypothesized to have an impact on cognition and other health outcomes, we observed only weak associations between cognitive function and sleep duration, sleep efficiency and WASO, in line with a previous study of 36 treated PWH [48].

Multivariate approaches such as PLS regression and random forest permit the investigation of a larger number of sleep characteristics as well as consideration of within-individual variability of these over a period of time, overcoming issues related to multicollinearity between sleep measures, multiple testing, and the estimation of large numbers of parameters. Here, both methods seem to indicate that longer nocturnal awakenings and greater within-individual variability of awakenings are associated with poorer cognitive function in PWH, with stronger associations than seen with any other sleep measure considered. Furthermore, other aspects related to the intraindividual variability of sleep patterns (e.g. the variability of sleep maintenance efficiency and out-of-bed time) appeared to have a negative effect on the overall cognitive function of PWH, with average sleep duration itself playing a marginal role. These are novel findings, given the limited number of studies that have investigated these aspects of sleep in relation to cognition even in the general population.

Of studies that have gone beyond sleep duration, several have shown similar results in demonstrating the potential deleterious cognitive impact of poor sleep continuity, as indicated by objectively measured WASO, sleep efficiency, or number of nocturnal awakenings, to which sleep duration appeared to add little [17, 20, 21]. Nevertheless, we have investigated a larger array of measures underlying the variability of sleep patterns than previous studies. Our findings suggesting the potential deleterious cognitive impact of high night-to-night variability of sleep efficiency, high variability of sleep fragmentation, and longer duration of nocturnal awakenings have not been comprehensively investigated previously and could result from important behavioral, social, and environmental factors as well as circadian or homeostatic drive. While these findings require further validation in other cohorts, it is possible that chronic disruptions to the circadian rhythm and homeostatic drive could affect the function and structure of brain regions such as the prefrontal cortex [49], responsible for high-order cognitive functions. Our study did not include formal assessments of circadian rhythm (e.g. dim light melatonin onset, core body temperature tracking) and an actigraphy-derived analysis of circadian rhythm was beyond the scope of this current analysis, but such future analyses will be important to a more comprehensive understanding of how sleep and circadian rhythms relate to cognition.

The use of a composite sleep score and predefined sleep dimensions failed to reveal any strong association between sleep and cognition in PWH, with only sleep efficiency showing a weak association that was close to reaching statistical significance. Finally, an individual-based approach such as LCA, which aims to identify homogenous groups of individuals with similar sleep profiles, also failed to detect any important relationships between objective sleep measures and cognition. While LCA identified two distinct groups of PWH distinguishing those with more irregular, fragmented, and inefficient sleep patterns from the rest of PWH, it showed no evident differences in the cognitive performance of the two groups.

Another important aim of our study was to assess whether the association between sleep measures and cognitive function in PWH differed from those seen in HIV-negative individuals with similar lifestyles. Of the approaches explored, we could formally compare associations by HIV status using the traditional approach (i.e. specific sleep characteristics using regression models) and the overall sleep score because PLS regression, random forest, and LCA do not allow assessment of interaction terms. In general, associations did not seem to differ between PWH and HIV-negative individuals. However, the direction of the association between cognitive function and sleep efficiency appeared to be significantly different in the two groups when using a categorical score reflecting the frequency of poor efficiency. While the possibility of a false-positive finding cannot be ruled out, the differential association may reflect different underlying causes of poor sleep efficiency in those with and without HIV, so further studies are warranted to better elucidate the effects of sleep efficiency on cognitive outcomes in PWH.

PLS regression and random forest highlight some similarities between the associations seen in PWH and HIV-negative individuals. In particular, the length of nocturnal awakenings and the within-individual variability in this measure seem to have a similarly important relationship to cognitive performance, regardless of HIV status. On the other hand, among HIV-negative individuals, aspects related to sleep fragmentation and overnight movement/limb motions are more strongly associated with cognition than among PWH. Possible explanations include altered clearance of toxins or disturbed sleep neurophysiology (e.g. reduced slow-wave sleep and rapid eye movement) reflected by greater sleep fragmentation causing less restorative sleep and leading, in turn, to deleterious effects on cognition.

By adopting a multimethod analytic approach, we comprehensively and robustly investigated the proposed associations beyond the limitations of each analytical method, while also highlighting the differences in the findings across methods. Each approach used has its own strengths as well as weaknesses (Table 1). The traditional approach may fail to detect associations of individual and often neglected sleep characteristics that are not selected when only a few characteristics are analyzed. PLS regression does not allow the incorporation of potential confounders into analyses and assumes a linear relationship between the sleep measures and the outcome of interest. Random forest does not provide a direct measure that would allow the evaluation of either the direction or magnitude of any associations. Moreover, variable importance metrics obtained from random forests are known to be biased when input variables are of different measurement scales and when are highly correlated [50]. Nevertheless, here we also reported another importance metric (i.e. the decrease in *R*^2^ due to the exclusion of a given variable from the model, Supplementary Figure 3) that showed results similar to the VIMs, in terms of the actigraphy variables which appear to more strongly contribute toward the prediction of cognitive scores. Multidimensional sleep constructs rely heavily on a priori conceptualizations of sleep health which may not be appropriate for a specific health outcome or a unique population such as PWH. Moreover, statistical power is reduced when continuous measures are dichotomized. LCA does not directly aim to evaluate associations with outcomes but rather aims to partition the population under study into groups based on the observed sleep characteristics, without regard to the outcome of interest; therefore, the obtained groups may not necessarily identify those at greatest risk of a poor outcome nor, in turn, the sleep characteristics that might predict this outcome.

Some limitations of our study need to be considered. Firstly, given the cross-sectional nature of the study, we cannot establish the direction or causal nature of any associations seen. Secondly, unmeasured confounding (e.g. physical exercise and stress) may have resulted in biased estimates of the associations of interest. Thirdly, PWH recruited in this study are mainly white men having sex with men, on effective HIV treatment with stable viral suppression, and therefore our results may not be generalizable to other populations of PWH with different sociodemographic and clinical characteristics, in particular women and PWH with poorly controlled HIV. Moreover, differences in terms of age and age-related factors between PWH and HIV-negative individuals may have introduced bias when comparing the relationship between sleep and cognition across the two groups. However, when possible, we included age and other potential confounders in the analysis so to minimize this potential bias. The sample size was pragmatically set considering resource and time constraints and was not based on the statistical power of any of the methods used. While our study is one of the largest in its fields, some methods, for example LCA, may have a lower power to detect significant associations than others, sample size being equal. Lastly, we used actigraphy-based assessments of sleep parameters rather than more detailed sleep measurements such as polysomnography. We therefore could not study detailed sleep physiology and mechanisms. Future studies incorporating polysomnogram data would likely benefit from machine learning approaches similar to our study, given the multidimensional and often collinear nature of polysomnogram data. We feel that such work with detailed physiologic measures and robust statistical approaches has tremendous potential to allow our field to better understand mechanistic pathways and to develop novel therapeutic interventions.

## Conclusions

Through the use of analytical approaches that allow the simultaneous consideration of multiple sleep characteristics, we found that aspects related to sleep continuity and regularity, including several novel measures of within-individual variability of wakefulness during sleep and sleep efficiency, were associated with cognitive performance in PWH. Other aspects of sleep more traditionally thought to be related to health problems (e.g. sleep duration) did not appear to have strong associations. Sleep is increasingly recognized as having a multidimensional construct, and our analysis demonstrates that multivariate analytical approaches can provide novel insights into the role of sleep on cognitive function and other health outcomes. A better understanding of which aspects of sleep are most strongly linked to a given outcome would help the development of targeted interventions to improve those aspects of sleep and, in turn, improve other health outcomes.

## Supplementary Material

zsab035_suppl_Supplementary_Materials
