# Supplementary material for: Sleep health and cognitive function among people with and without HIV: the use of different machine learning approaches
Source: Sleep. 2021 Feb 16;44(8):zsab035. doi: 10.1093/sleep/zsab035 (PMC8361343; doi:10.1093/sleep/zsab035)
Supplement: zsab035_suppl_Supplementary_Materials [file zsab035_suppl_supplementary_materials.docx]

# Sleep health and cognitive function among people with and without HIV: the use of different machine learning approaches

**Supplementary Material**

Davide De Francesco (1), Caroline A. Sabin (1), Alan Winston (2), Michael N Rueschman (3, 4), Nicki D. Doyle (2), Jane Anderson (5), Jaime H. Vera (6), Marta Boffito (7), Memory Sachikonye (8), Patrick W.G. Mallon (9), Lewis Haddow (1, 10), Frank A. Post (11), Susan Redline (3, 4, 12), Ken M. Kunisaki (13, 14)

1. Institute for Global Health, University College London, London, UK;
2. Department of Infectious Disease, Imperial College London, London, UK;
3. Brigham and Women's Hospital, Boston, USA;
4. Harvard Medical School, Harvard University, Boston, USA;
5. Homerton University Hospital, London, UK;
6. Brighton and Sussex Medical School, Brighton, UK;
7. Chelsea and Westminster Healthcare NHS Foundation Trust, London, UK;
8. UK Community Advisory Board (UK-CAB), London, UK;
9. University College Dublin School of Medicine, Dublin, Ireland;
10. Kingston Hospital NHS Foundation Trust, London, UK;
11. King's College Hospital NHS Foundation Trust, London, UK;
12. Beth Israel Deaconess Medical Center, Boston, USA;
13. Minneapolis Veterans Affairs Health Care System, Minneapolis, USA;
14. University of Minnesota, Minneapolis, USA.

## Corresponding author

Prof. Caroline A. Sabin

Institute for Global Health, UCL, Royal Free Campus, Rowland Hill Street, London, NW3 2PF.

Email: [c.sabin@ucl.ac.uk](mailto:c.sabin@ucl.ac.uk); Telephone: +44 207 7940500 ext. 34752

## Supplementary methods

Partial least squares (PLS) regression

As PLS regression works best for set of correlated and homogeneous continuous variables, only actigraphy measures were selected.

The number of predictive components that we aimed to extract was determined based on the proportion of variation in the global T-score explained by the model. Extraction of components (up to a maximum of ten) stopped when the proportion of variation explained (R^2^) by the additional component was <1%. The R^2^ for each model was assessed using 10-fold cross-validation. Briefly, the data were partitioned into 10 mutually exclusives groups and, one at a time, each group was omitted from the calculation of model parameters. Global T-scores were predicted for each group of observations using the model calculated on the remaining nine groups and the cross-validated R^2^ and mean squared error (MSE) were calculated.

The PLS regression model obtained in people with HIV (PWH) achieved a R^2^ of 0.09 and a MSE of 58.5. R^2^ and MSE for the final PLS regression model in HIV-negative individuals were 0.19 and 34.4, respectively.

Random forest

Hyper-parameters such as the number of decision trees in the forest and the number of features considered by each tree were optimized evaluating all possible combinations, with the number of trees varying from 1000 to 10000 (with increments of 200 units) and the number of features varying from 5 to 20. 10-fold cross-validated R^2^ and MSE were calculated for each of these combinations (see figure below) and the combination with the best R^2^ and lowest MSE was adopted.

Among PWH, the model with 5000 trees and 15 features achieved an R^2^ of 0.25 and an MSE of 47.0. Among HIV-negative individuals, the model with the best R^2^ and lowest MSE was the one with 8200 trees and 5 features; R^2^ and MSE from this model were 0.07 and 39.7, respectively (Supplementary Figure 1).


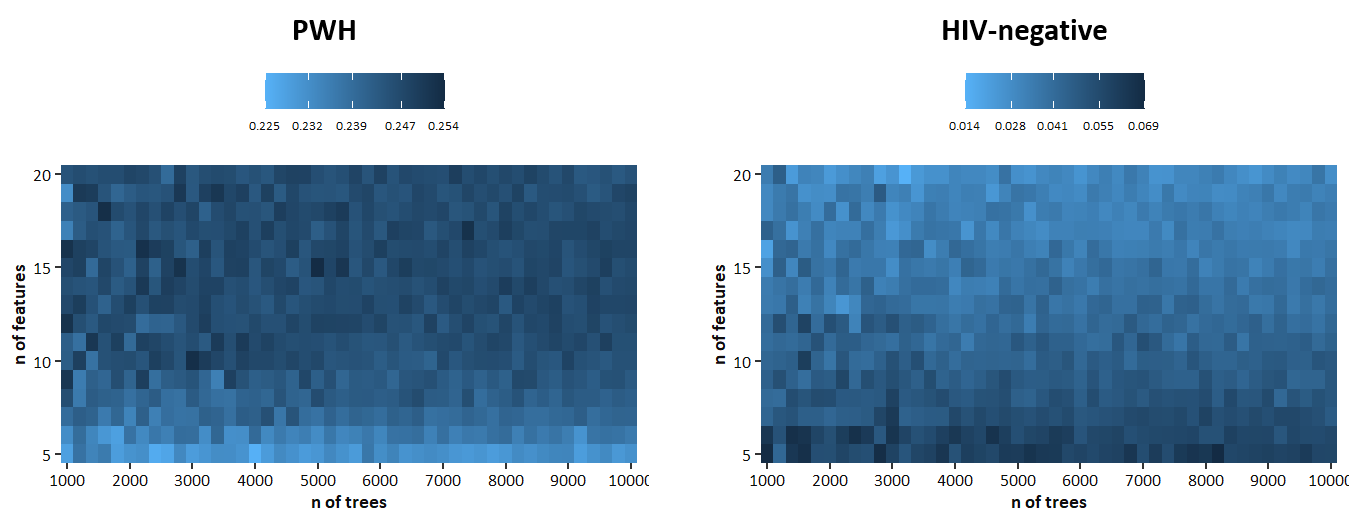


**Supplementary Figure 1:** R^2^ for different random forests depending on the number of trees and the number of features selected within each tree.

The raw variable importance measure (VIM) for each variable (including actigraphy measures and potential confounders) are shown below (Supplementary Figure 2). Please note that, in the manuscript, VIM for actigraphy measures only are reported and are expressed as the percentage relative to that of the actigraphy variable with the highest VIM.

In addition, Supplementary Figure 3 shows the decrease in R^2^ due to the exclusion of each variable (both actigraphy measures and potential confounders) from the model including all the other variables. Among PWH, the actigraphy variables resulting in the greater decrease in R^2^ were SD of out-of-bed time, average number and mean length of awakenings, SD of the number and mean length of awakenings, and SD of out-of-bed time. Among HIV-negative individuals, SD and average of maintenance efficiency, average movement index, SD of number and mean length of awakenings were the actigraphy variables associated with the greatest decrease in R^2^.


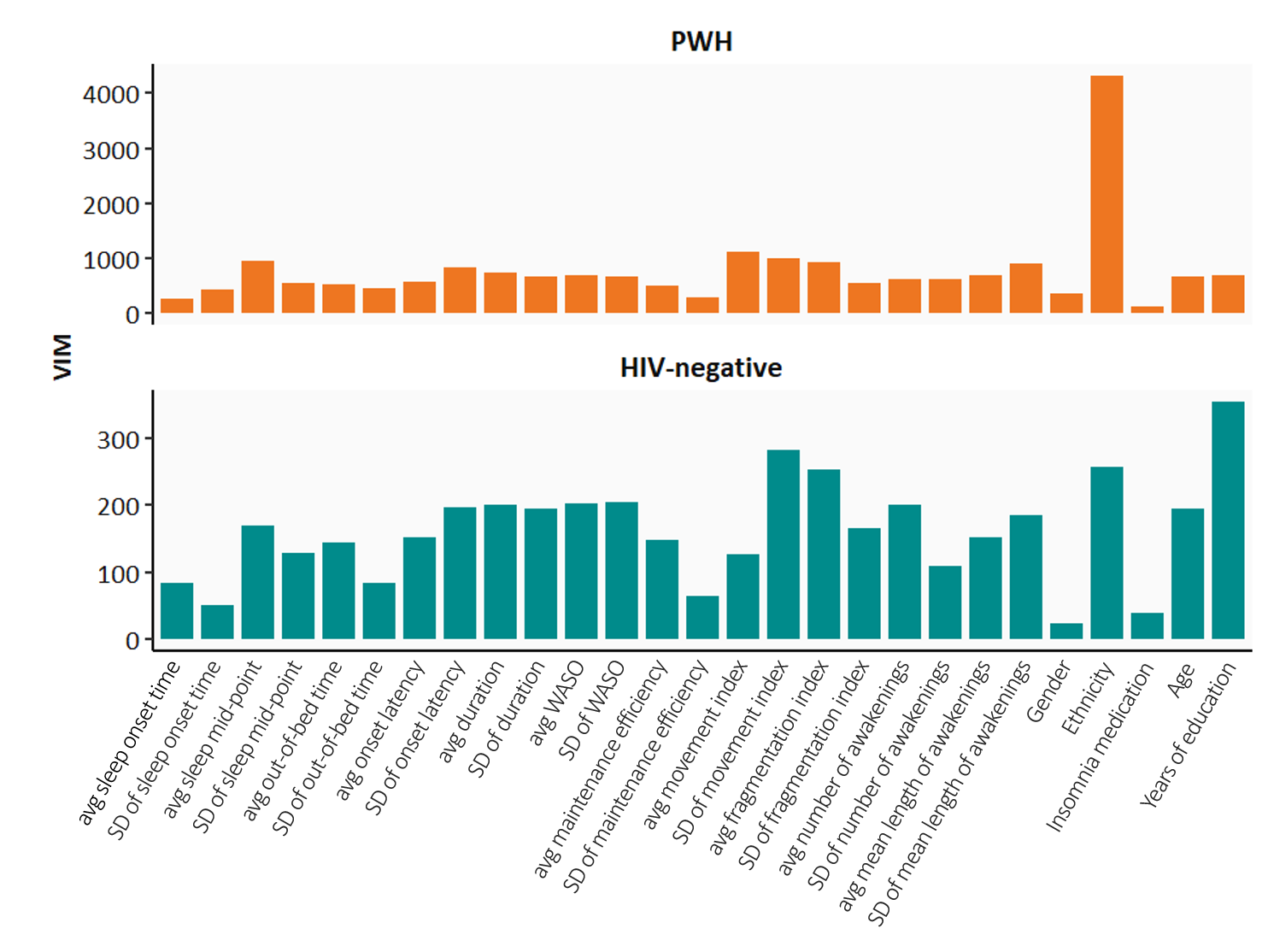


**Supplementary Figure 2:** VIM for each variable according to random forest. VIM indicates the total decrease in the residual sum of squares from splitting on that variable, averaged over all trees. The higher the VIM the stronger is the association between the variable and the outcome.


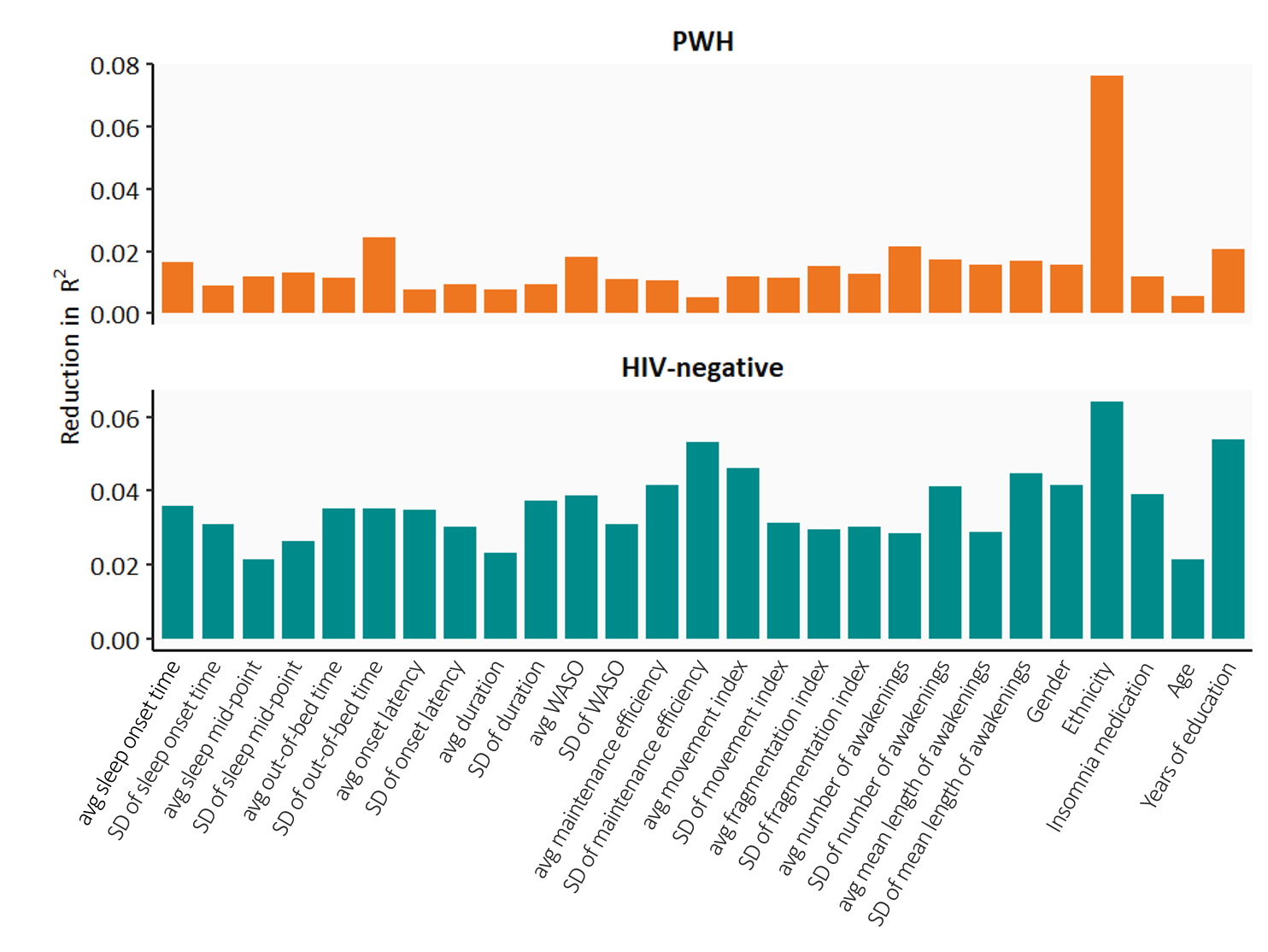


**Supplementary Figure 3:** Decrease in R^2^ due to the exclusion of each variable from the model including all the other variables.

Latent class analysis (LCA)

The model-based approach to clustering used allows the specification of different configurations based on the number of clusters and three parameters of the multivariate parametric distribution of latent space underlying actigraphy measures for each of the clusters identified. These three parameters are the distribution (spherical or ellipsoidal), volume (equal or variable) and shape (equal or variable) of the covariances, each of which can be constrained to be equal or variable across clusters. Thus, for a given number of clusters, 7 possible models with different geometric characteristics can be specified. We explored 7x9 models, as the number of clusters was set to vary between 2 and 10. The optimal model, in terms of both number of clusters as well as distribution, volume and shape of the covariances, was selected using the Bayesian Information Criterion (BIC). After selection of the model with the best fit out of the 7 models tested, a bootstrap likelihood ratio test was performed to establish whether a model with an additional cluster to the optimal number of clusters indicated by the BIC, fitted the data significantly better than the model with the number of cluster as indicated by the BIC. Parametric bootstrap with 1,000 replications was used.

For both PWH and HIV-negative individuals, the model with two clusters and with ellipsoidal distribution, equal volume and equal shape obtained the lowest BIC and, therefore, the best fit (see Supplementary Figure 4). According to the bootstrap likelihood ratio test, the model with three clusters did not improve model fit in both PWH (likelihood ratio test statistic = 45.35, p=0.73) and HIV-negative individuals (likelihood ratio test statistic = 38.17, p=0.78).


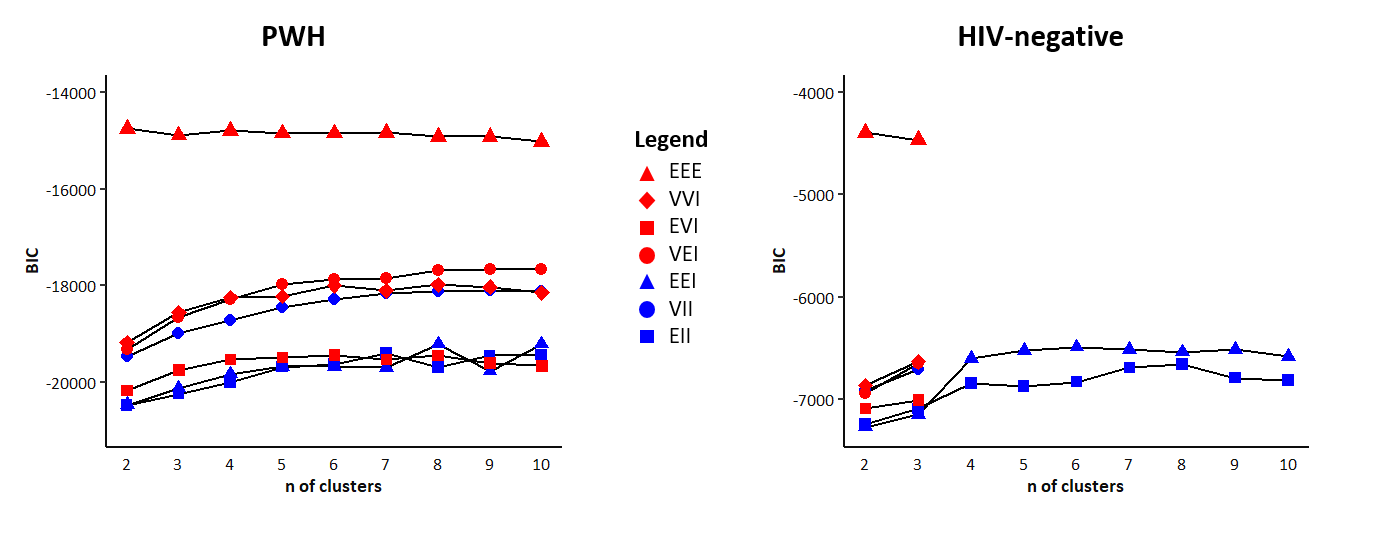


**Supplementary Figure 4:** BIC for different numbers of clusters and with different assumptions regarding the volume, shape and orientation of covariances between input features. Due to singularity in the covariance matrix estimate some models could not be estimated. EEE= ellipsoidal distribution, equal volume and equal shape, VVI = diagonal distribution, variable volume and variable shape, EVI = diagonal distribution, equal volume and variable shape, VEI = diagonal distribution, variable volume and equal shape, EEI = diagonal distribution, equal volume and equal shape, VII = spherical distribution, variable volume and equal shape, EII = spherical distribution, equal volume and equal shape.

Stability measure were computed, evaluating the consistency of a clustering result by comparing it with the clusters obtained after each variable is removed, one at a time. These included the average proportion of non-overlap (APN), the average distance (AD), the average distance between means (ADM), and the figure of merit (FOM). The optimal clustering result is the one which minimizes all these stability measures. As shown in Supplementary Figure 5, for both PWH and HIV-negative individuals, the model with two cluster gave the best scores in terms of APN and ADM, whereas the model with 10 clusters showed the best FOM and AD scores.


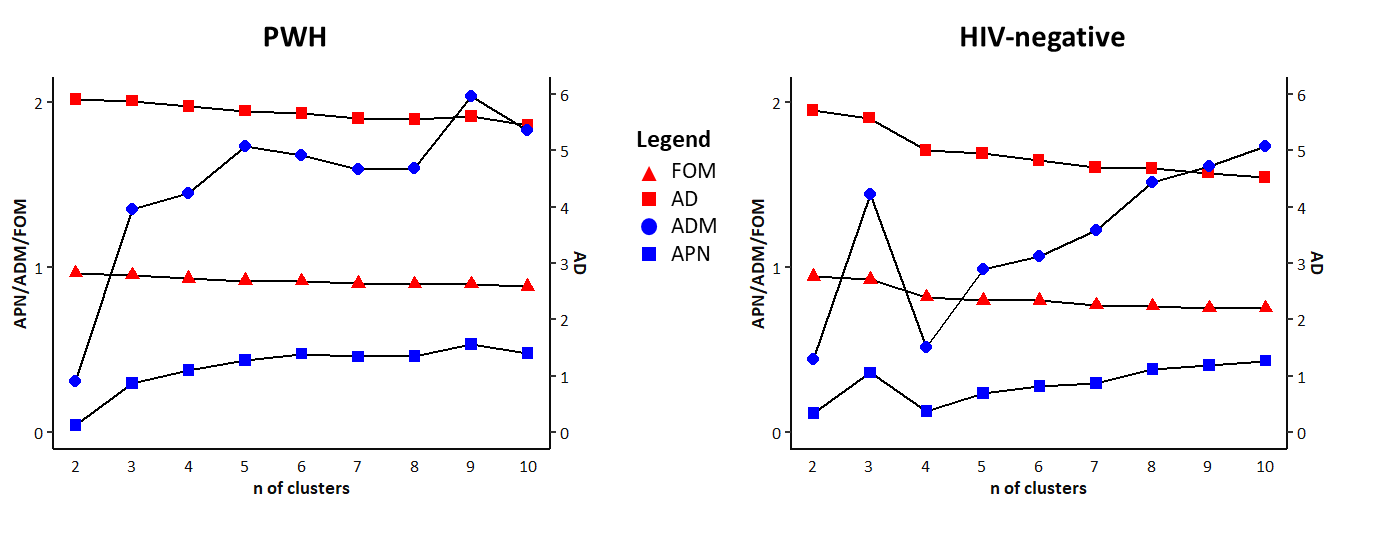


**Supplementary Figure 5:** Average proportion of non-overlap (APN), average distance (AD), average distance between means (ADM) and figure of merit (FOM) for different numbers of clusters.

**Supplementary Table 1:** Individual tests of the cognitive battery and how they map into cognitive domains.

| **Cognitive domain** | **Test** | **Scoring system** |
| --- | --- | --- |
| Attention | PASAT 3 | Total correct summations |
| Executive function | Trail Making Test-B | Total time to complete |
| Language | Category Fluency | Total number of animals in 1 minute |
|  | Letter Fluency | Total number of words, 1 minute for each of 3 letters |
| Motor function | Grooved pegboard | Dominant hand: Time to complete |
|  | Grooved pegboard | Non-dominant hand: Time to complete |
| Processing speed | Trail Making Test-A | Time to complete |
|  | WAIS-III Digit Symbol | Total correct symbols |
|  | WAIS-III Symbol Search | Total correct symbols |
|  | Stroop colour-word test | Number of items completed |

Note: PASAT: Paced auditory serial addition test; WAIS: Wechsler adult intelligence scale

**Supplementary Table 2:** Median (IQR) of cognitive T-scores and sleep actigraphy measures in PWH and HIV-negative individuals; p-values were estimated using median regression accounting for age

| Median (IQR) or n (%) | PWH (n=344) | HIV-negative (n=119) | p-value |
| --- | --- | --- | --- |
| **Cognitive scores** |  |  |  |
| Global T-score | 50.0 (44.2, 54.9) | 52.0 (48.3, 55.6) | 0.21 |
| Language T-score | 50.2 (43.3, 58.1) | 52.5 (48.1, 59.1) | 0.38 |
| Attention T-score | 45.5 (34.1, 53.6) | 44.4 (30.0, 53.7) | 0.99 |
| Processing speed T-score | 50.9 (45.0, 55.7) | 54.0 (48.4, 57.8) | 0.04 |
| Executive Function T-score | 52.2 (43.4, 60.1) | 55.2 (49.4, 62.9) | 0.40 |
| Motor function T-score | 50.7 (42.6, 57.3) | 51.5 (43.4, 58.1) | 0.25 |
| **Sleep measures** |  |  |  |
| Average sleep onset time [clock time] | 23:07 (21:22, 1:01) | 23:19 (22:38, 00:36) | 0.64 |
| SD of sleep onset time [minutes] | 50 (31, 78) | 37 (28, 61) | 0.05 |
| Average sleep mid-point [clock time] | 3:55 (3:07, 4:43) | 3:30 (2:58, 4:04) | 0.01 |
| SD of sleep mid-point [minutes] | 43 (29, 64) | 38 (25, 57) | 0.74 |
| Average out-of-bed time [clock time] | 7:53 (7:01, 8:48) | 7:29 (6:52, 8:11) | 0.02 |
| SD of out-of-bed time [minutes] | 57 (37, 80) | 48 (34, 67) | 0.53 |
| Average onset latency [minutes] | 7 (6, 9) | 7 (6, 9) | 1.00 |
| SD of onset latency [minutes] | 3 (2, 5) | 3 (2, 5) | 1.00 |
| Average duration [hours] | 7.2 (6.7, 7.6) | 7.0 (6.3, 7.6) | 0.88 |
| SD of duration [minutes] | 59 (40, 84) | 47 (36, 65) | 0.01 |
| Average WASO [minutes] | 54 (40, 74) | 49 (35, 69) | 0.85 |
| SD of WASO [minutes] | 20 (14, 29) | 16 (11, 25) | 0.20 |
| Average maintenance efficiency [%] | 88.7 (84.4, 91.4) | 90.2 (86.2, 92.2) | 0.07 |
| SD of maintenance efficiency [%] | 3.7 (2.6, 5.1) | 3.1 (2.2, 4.7) | 0.11 |
| Average movement index [%] | 17.7 (14.3, 22.5) | 15.2 (12.5, 19.6) | 0.003 |
| SD of movement index [%] | 3.6 (2.6, 5.2) | 2.9 (2.1, 4.2) | 0.04 |
| Average fragmentation index [%] | 30.6 (25.2, 37.8) | 28.8 (21.5, 34.1) | 0.32 |
| SD of fragmentation index [%] | 9.8 (7.6, 12.9) | 9.1 (7.1, 11.9) | 0.07 |
| Average number of awakenings | 19 (14, 23) | 16 (12, 22) | 0.59 |
| SD of number of awakenings | 5 (4, 7) | 5 (3, 6) | 0.47 |
| Average mean length of awakenings [min] | 3.1 (2.5, 3.7) | 3.0 (2.5, 3.7) | 0.08 |
| SD of mean length of awakenings [min] | 0.8 (0.6, 1.2) | 0.8 (0.6, 1.2) | 0.08 |
| RU SATED score | 7 (6, 9) | 8 (7, 10) | <0.001 |

**Supplementary Table 3:** Spearman’s correlation coefficient between actigraphy variables in PWH and HIV-negative individuals (combined); p-values were adjusted using the Bonferroni correction

|  | 2. | 3. | 4. | 5. | 6. | 7. | 8. | 9. | 10. | 11. | 12. |
| --- | --- | --- | --- | --- | --- | --- | --- | --- | --- | --- | --- |
| 1. avg onset latency | 0.84 (p=<0.001) | -0.15 (p=0.001) | 0.07 (p=1.00) | 0.26 (p=0.01) | 0.21 (p=0.20) | -0.28 (p=0.003) | 0.24 (p=0.09) | 0.28 (p=<0.001) | 0.25 (p=0.21) | 0.22 (p=0.05) | 0.01 (p=1.00) |
| 2. SD of onset latency |  | -0.09 (p=0.01) | 0.08 (p=1.00) | 0.24 (p=0.19) | 0.19 (p=1.00) | -0.24 (p=0.09) | 0.19 (p=0.87) | 0.25 (p=0.03) | 0.25 (p=1.00) | 0.19 (p=1.00) | 0.00 (p=1.00) |
| 3. avg duration |  |  | -0.01 (p=1.00) | -0.07 (p=1.00) | -0.09 (p=0.73) | 0.32 (p=0.65) | -0.27 (p=0.06) | -0.19 (p=0.14) | -0.22 (p=0.03) | -0.14 (p=1.00) | 0.00 (p=1.00) |
| 4. SD of duration |  |  |  | 0.17 (p=1.00) | 0.32 (p=1.00) | -0.18 (p=1.00) | 0.29 (p=1.00) | 0.23 (p=1.00) | 0.37 (p=0.23) | 0.12 (p=1.00) | 0.01 (p=1.00) |
| 5. avg WASO |  |  |  |  | 0.70 (p=<0.001) | -0.96 (p=<0.001) | 0.59 (p=<0.001) | 0.72 (p=<0.001) | 0.43 (p=0.02) | 0.55 (p=<0.001) | -0.17 (p=1.00) |
| 6. SD of WASO |  |  |  |  |  | -0.67 (p=<0.001) | 0.87 (p=<0.001) | 0.59 (p=<0.001) | 0.64 (p=<0.001) | 0.44 (p=0.002) | 0.03 (p=1.00) |
| 7. avg maintenance efficiency |  |  |  |  |  |  | -0.64 (p=<0.001) | -0.73 (p=<0.001) | -0.46 (p=0.01) | -0.57 (p=<0.001) | 0.15 (p=1.00) |
| 8. SD of maintenance efficiency |  |  |  |  |  |  |  | 0.55 (p=<0.001) | 0.71 (p=<0.001) | 0.43 (p=0.005) | 0.09 (p=1.00) |
| 9. avg movement index |  |  |  |  |  |  |  |  | 0.62 (p=<0.001) | 0.58 (p=<0.001) | -0.05 (p=1.00) |
| 10. SD of movement index |  |  |  |  |  |  |  |  |  | 0.36 (p=0.09) | 0.11 (p=1.00) |
| 11. avg fragmentation index |  |  |  |  |  |  |  |  |  |  | 0.20 (p=1.00) |
| 12. SD of fragmentation index |  |  |  |  |  |  |  |  |  |  |  |
| 13. avg number of awakenings |  |  |  |  |  |  |  |  |  |  |  |

**Supplementary Table 3 (continued)**

|  | 13. | 14. | 15. | 16. | 17. | 18. | 19. | 20. | 21. | 22. |
| --- | --- | --- | --- | --- | --- | --- | --- | --- | --- | --- |
| 1. avg onset latency | 0.13 (p=1.00) | 0.10 (p=1.00) | 0.20 (p=0.06) | 0.17 (p=1.00) | -0.04 (p=1.00) | 0.10 (p=1.00) | 0.07 (p=1.00) | 0.06 (p=1.00) | 0.05 (p=1.00) | 0.08 (p=1.00) |
| 2. SD of onset latency | 0.12 (p=1.00) | 0.08 (p=1.00) | 0.22 (p=0.04) | 0.19 (p=1.00) | -0.02 (p=1.00) | 0.08 (p=1.00) | 0.10 (p=1.00) | 0.04 (p=1.00) | 0.09 (p=1.00) | 0.08 (p=1.00) |
| 3. avg duration | 0.06 (p=1.00) | 0.06 (p=1.00) | -0.16 (p=<0.001) | -0.12 (p=0.01) | 0.18 (p=1.00) | -0.08 (p=1.00) | 0.01 (p=1.00) | -0.07 (p=1.00) | 0.35 (p=1.00) | -0.09 (p=1.00) |
| 4. SD of duration | 0.09 (p=1.00) | 0.31 (p=1.00) | 0.16 (p=1.00) | 0.18 (p=1.00) | -0.02 (p=1.00) | 0.60 (p=<0.001) | 0.22 (p=0.60) | 0.61 (p=<0.001) | 0.20 (p=1.00) | 0.66 (p=<0.001) |
| 5. avg WASO | 0.78 (p=<0.001) | 0.49 (p=<0.001) | 0.49 (p=1.00) | 0.10 (p=1.00) | 0.09 (p=1.00) | 0.24 (p=1.00) | 0.00 (p=1.00) | 0.16 (p=1.00) | 0.13 (p=1.00) | 0.14 (p=1.00) |
| 6. SD of WASO | 0.49 (p=0.02) | 0.63 (p=<0.001) | 0.41 (p=1.00) | 0.32 (p=1.00) | 0.06 (p=1.00) | 0.36 (p=1.00) | 0.01 (p=1.00) | 0.30 (p=1.00) | 0.08 (p=1.00) | 0.29 (p=1.00) |
| 7. avg maintenance efficiency | -0.72 (p=0.001) | -0.43 (p=0.005) | -0.51 (p=1.00) | -0.12 (p=1.00) | -0.05 (p=1.00) | -0.25 (p=1.00) | 0.00 (p=1.00) | -0.17 (p=1.00) | -0.04 (p=1.00) | -0.16 (p=1.00) |
| 8. SD of maintenance efficiency | 0.38 (p=0.34) | 0.52 (p=0.02) | 0.39 (p=0.20) | 0.38 (p=1.00) | 0.02 (p=1.00) | 0.33 (p=1.00) | 0.02 (p=1.00) | 0.28 (p=1.00) | 0.00 (p=1.00) | 0.26 (p=1.00) |
| 9. avg movement index | 0.55 (p=0.002) | 0.44 (p=0.002) | 0.37 (p=1.00) | 0.16 (p=1.00) | 0.04 (p=1.00) | 0.27 (p=1.00) | 0.08 (p=1.00) | 0.21 (p=1.00) | 0.11 (p=1.00) | 0.19 (p=1.00) |
| 10. SD of movement index | 0.21 (p=1.00) | 0.41 (p=0.06) | 0.36 (p=0.18) | 0.36 (p=1.00) | 0.01 (p=1.00) | 0.39 (p=0.19) | 0.09 (p=1.00) | 0.33 (p=1.00) | 0.08 (p=1.00) | 0.29 (p=0.38) |
| 11. avg fragmentation index | 0.52 (p=<0.001) | 0.35 (p=0.01) | 0.16 (p=1.00) | -0.01 (p=1.00) | 0.08 (p=1.00) | 0.21 (p=1.00) | 0.02 (p=1.00) | 0.13 (p=1.00) | 0.05 (p=1.00) | 0.11 (p=1.00) |
| 12. SD of fragmentation index | -0.30 (p=1.00) | -0.06 (p=1.00) | 0.16 (p=1.00) | 0.24 (p=1.00) | -0.03 (p=1.00) | 0.05 (p=1.00) | 0.05 (p=1.00) | 0.05 (p=1.00) | 0.01 (p=1.00) | 0.01 (p=1.00) |
| 13. avg number of awakenings |  | 0.59 (p=<0.001) | -0.09 (p=1.00) | -0.33 (p=1.00) | 0.10 (p=1.00) | 0.21 (p=1.00) | 0.02 (p=1.00) | 0.15 (p=1.00) | 0.18 (p=1.00) | 0.08 (p=1.00) |

**Supplementary Table 3 (continued)**

|  | 15. | 16. | 17. | 18. | 19. | 20. | 21. | 22. |
| --- | --- | --- | --- | --- | --- | --- | --- | --- |
| 14. SD of number of awakenings | -0.02 (p=1.00) | -0.06 (p=1.00) | 0.02 (p=1.00) | 0.36 (p=0.09) | 0.07 (p=1.00) | 0.33 (p=0.39) | 0.13 (p=1.00) | 0.27 (p=1.00) |
| 15. avg mean length of awakenings |  | 0.70 (p=<0.001) | 0.02 (p=1.00) | 0.08 (p=1.00) | 0.01 (p=1.00) | 0.04 (p=1.00) | 0.02 (p=1.00) | 0.09 (p=1.00) |
| 16. SD of mean length of awakenings |  |  | -0.04 (p=1.00) | 0.10 (p=1.00) | 0.09 (p=1.00) | 0.09 (p=1.00) | 0.04 (p=1.00) | 0.13 (p=1.00) |
| 17. avg sleep onset time |  |  |  | -0.04 (p=1.00) | -0.39 (p=0.03) | -0.05 (p=1.00) | -0.24 (p=1.00) | -0.06 (p=1.00) |
| 18. SD of sleep onset time |  |  |  |  | 0.23 (p=1.00) | 0.77 (p=<0.001) | 0.21 (p=1.00) | 0.43 (p=<0.001) |
| 19. avg sleep mid-point |  |  |  |  |  | 0.24 (p=0.62) | 0.86 (p=0.29) | 0.22 (p=0.91) |
| 20. SD of sleep mid-point |  |  |  |  |  |  | 0.22 (p=1.00) | 0.80 (p=<0.001) |
| 21. avg out-of-bed time |  |  |  |  |  |  |  | 0.18 (p=1.00) |
| 22. SD of out-of-bed time |  |  |  |  |  |  |  |  |

**Supplementary Table 4:** Number and proportion of PWH and HIV-negative individuals reporting good, fair and poor health in each of the six RU SATED sleep dimensions; p-values were estimated using Chi-square or Fisher’s exact test, as appropriate

|  | PWH (n=344) | HIV-negative (n=119) | p-value |
| --- | --- | --- | --- |
| **Satisfaction** |  |  | 0.008 |
| Good | 79 (23.2%) | 44 (37.0%) |  |
| Fair | 156 (45.7%) | 50 (42.0%) |  |
| Poor | 106 (31.0%) | 25 (21.0%) |  |
| **Alertness** |  |  | 0.07 |
| Good | 255 (74.3%) | 100 (84.8%) |  |
| Fair | 64 (18.7%) | 13 (11.0%) |  |
| Poor | 24 (7.0%) | 5 (4.2%) |  |
| **Timing** |  |  | <0.001 |
| Good | 295 (86.2%) | 117 (98.3%) |  |
| Fair | 31 (9.1%) | 2 (1.7%) |  |
| Poor | 16 (4.7%) | 0 (0.0%) |  |
| **Efficiency** |  |  | 0.07 |
| Good | 189 (55.3%) | 78 (65.6%) |  |
| Fair | 68 (19.9%) | 23 (19.3%) |  |
| Poor | 85 (24.9%) | 18 (15.1%) |  |
| **Duration** |  |  | 0.001 |
| Good | 107 (31.3%) | 59 (49.6%) |  |
| Fair | 139 (40.6%) | 39 (32.8%) |  |
| Poor | 96 (28.1%) | 21 (17.6%) |  |
| **Regularity** |  |  | 0.05 |
| Good | 90 (26.3%) | 35 (29.4%) |  |
| Fair | 21 (6.1%) | 1 (0.8%) |  |
| Poor | 231 (67.5%) | 83 (69.8%) |  |
